# NIA OFFICE OF SPECIAL POPULATIONS

**DOI:** 10.1093/geroni/igae098.0336

**Published:** 2024-12-31

**Authors:** Patricia Jones

**Affiliations:** National Institute on Aging, Baltimore, Maryland, United States

## Abstract

Dr. Jones will discuss the work of the NIA Office of Special Populations. Dr. Jones will also be available for small group discussion.

